# Multidrug-resistant Strains of *Salmonella* *enterica* Typhimurium, United States, 1997–1998^1^

**DOI:** 10.3201/eid1005.030209

**Published:** 2004-05

**Authors:** Therese Rabatsky-Ehr, Jean Whichard, Shannon Rossiter, Ben Holland, Karen Stamey, Marcia L. Headrick, Timothy J. Barrett, Frederick J. Angulo, the NARMS Working Group

**Affiliations:** * Yale University School of Medicine, New Haven, Connecticut, USA; † Centers for Disease Control and Prevention, Atlanta, Georgia, USA; ‡ U.S. Food and Drug Administration, Bethesda, Maryland, USA

**Keywords:** *Salmonella* Typhimurium, National Antimicrobial Resistance Monitoring System, NARMS, CDC, phage type, DT104, drug resistance-multiple, public health, epidemiology, United States

## Abstract

To evaluate multidrug-resistant strains of *Salmonella* *enterica* Typhimurium, including definitive type 104 (DT104) in the United States, we reviewed data from the National Antimicrobial Resistance Monitoring System (NARMS). In 1997–1998, 25% (703) of 2,767 serotyped *Salmonella* isolates received at NARMS were *S*. Typhimurium; antimicrobial susceptibility testing and phage typing were completed for 697. Fifty-eight percent (402) were resistant to >1 antimicrobial agent. Three multidrug-resistant (>5 drugs) strains accounted for 74% (296) of all resistant isolates. Ceftriaxone resistance was present in 3% (8), and nalidixic acid resistance in 1% (4), of these multidrug-resistant strains. By phage typing, 37% (259) of *S*. Typhimurium isolates were DT104, 30% (209) were of undefined type and 15% (103) were untypable. Fifty percent (202) of resistant (>1 drug) isolates were DT104. Multidrug-resistant *S*. Typhimurium isolates, particularly DT104, account for a substantial proportion of *S*. Typhimurium isolates; ceftriaxone resistance is exhibited by some of these strains.

*Salmonella enterica* serotype Typhimurium is the most common *Salmonella* serotype in the United States, accounting for 29% of the approximately 30,000 laboratory-confirmed *Salmonella* infections reported annually to the Centers for Disease Control and Prevention (CDC) from 1968 to 1998 (*1*). Among *Salmonella* serotypes, Typhimurium exhibits one of the highest prevalences of antimicrobial resistance (*2*,*3*). Of particular concern is a multidrug-resistant strain of *S*. Typhimurium defined by phage typing as definitive type 104 (DT104). Multidrug-resistant DT104 was first detected in the United Kingdom in 1984 and was first isolated in the United States in 1985 (*4*,*5*). In addition to the phage reactions, this strain is characterized by its multiple antimicrobial-resistance pattern to ampicillin, chloramphenicol, streptomycin, sulfamethoxazole, and tetracycline (R-type ACSSuT). The number of reported human isolates of DT104 R-type ACSSuT in the United Kingdom increased from 259 isolates in 1990 to 4,006 isolates in 1996 (*6*).

In the United States, antimicrobial susceptibility testing determined that the proportion of *S*. Typhimurium isolates that were R-type ACSSuT increased from <1% in 1980 to 34% in 1996 (*7*). Although phage typing of *S*. Typhimurium isolates is not routinely done in the United States, 93% of the R-type ACSSuT isolates tested from a national sample of isolates from all state and public health laboratories conducted in 1995 were DT104, which suggests that 9% of all human *Salmonella* infections in this country in 1995 were caused by *S*. Typhimurium DT104 R-type ACSSuT (*5*,*7*).

The objectives of this analysis were to determine the antimicrobial-resistance patterns seen among *S*. Typhimurium isolates received at CDC through the National Antimicrobial Resistance Monitoring System (NARMS) from 1997 through 1998 and describe the distribution of phage types, including DT104, among *S*. Typhimurium isolates with the most common resistance patterns.

## Methods

In 1996, NARMS was established to prospectively monitor the patterns of antimicrobial-drug resistance among human enteric pathogens, including nontyphoidal *Salmonella* isolates received at select public health laboratories in the United States (*8*). NARMS began as collaboration between CDC, the U.S. Food and Drug Administration Center for Veterinary Medicine, and 12 state health departments (California, Colorado, Connecticut, Florida, Georgia, Kansas, Massachusetts, Minnesota, New Jersey, Oregon, Washington, and West Virginia) and two local health departments (Los Angeles County and New York City). Two additional state health departments (Maryland and New York) joined NARMS in 1997. According to 1998 U.S. postcensus estimates (available from: http://www.census.gov/population/estimates/states), the population served by these 16 state and local health departments was approximately 97 million persons, 37% of the U.S. population.

From 1997 through 1998, NARMS-participating public health laboratories forwarded every 10th nontyphoidal *Salmonella* isolate serotyped at their laboratory to CDC for susceptibility testing. At CDC, partial range MICs were determined by using broth microdilution (Sensititre, Trek Diagnostics, Westlake, OH) for 16 antimicrobial agents: amikacin, amoxicillin-clavulanic acid (Cl), ampicillin (A), apramycin (Ap), ceftiofur (a third-generation cephalosporin used in veterinary medicine) (Cef), ceftriaxone (Cx), cephalothin (Cep), chloramphenicol (C), ciprofloxacin, gentamicin (G), kanamycin (K), nalidixic acid (N), streptomycin (S), sulfamethoxazole (Su), tetracycline (T), and trimethoprim-sulfamethoxazole (Tm) (NARMS 1997, 1998 Annual Reports; available from: http://www.cdc.gov/ncidod/dbmd/narms). National Committee for Clinical Laboratory Standards (NCCLS) interpretive criteria were used when available (*9*); resistance to ceftiofur, apramycin, and streptomycin was defined as an MIC ≥8 μg/mL, ≥32 μg/mL, and ≥64 μg/mL, respectively. Isolates that exhibited decreased susceptibility to third-generation cephalosporins (ceftiofur or ceftriaxone) were confirmed as *Salmonella* and tested for the full range of MICs for ceftriaxone by broth microdilution using NCCLS standards and by further molecular characterization (*10*,*11*). All isolates with intermediate susceptibilities were categorized as susceptible for this analysis with the understanding that an intermediate susceptibility to some drugs, in particular, ceftriaxone, would remove this drug as a clinical option.

Phage types were determined by using a scheme of 31 *S*. Typhimurium typing phages based on the method of Anderson et al. (*12*) and the interpretive guide supplied by the Public Health Laboratory Service (PHLS) in Colindale, United Kingdom. At the time this set of *S*. Typhimurium isolates was tested, additional *S*. Typhimurium typing phages 1, 2, 3, and 18 (which would have enabled designation of definitive types 193, 194, 195, and 208) were not used. *S*. Typhimurium isolates phage typed as definitive type 104, 104a, 104b, 104c, or U302 (closely related definitive types) were classified together as DT104 complex (hereafter referred to as DT104). Those *S*. Typhimurium isolates that reacted to phages but did not conform to any defined pattern were classified as RDNC (reacts but does not conform), and those that did not react with any of the typing phages used at the time were classified as untypable. Isolates that did not have antimicrobial susceptibility test results or phage type results were excluded from analysis. Invasive isolates were classified as those isolated from specimens collected from normally sterile sites, such as blood or cerebral spinal fluid; enteric isolates were those isolated from stool specimens or rectal swabs. Isolates from specimens collected from other (e.g., urine) or unknown sources were excluded from analysis by specimen source. Statistical analysis was performed with Epi Info 6.04 (CDC, Atlanta, GA) and SAS 6.12 software (SAS Institute Inc., Cary, NC). Statistical testing of differences in proportions was conducted using the chi-square test; p values <0.05 were considered significant.

## Results

### Resistance Testing

A total of 2,767 serotyped nontyphoidal *Salmonella* isolates were received at CDC through NARMS from 1997 through 1998; 1,301 in 1997 and 1,466 in 1998. Of these, 703 (25%) were *Salmonella* serotype Typhimurium (including serotype Typhimurium var. Copenhagen); 326 (25%) in 1997 and 377 (26%) in 1998. Antimicrobial susceptibility testing and phage typing was completed on 697 isolates.

The antimicrobial agents to which *S*. Typhimurium isolates demonstrated the highest level of resistance were sulfamethoxazole (53%), streptomycin (51%), tetracycline (50%), ampicillin (48%), chloramphenicol (35%), kanamycin (16%), amoxicillin-clavulanic acid (5%), cephalothin (5%), gentamicin (4%), trimethoprim-sulfamethoxazole (4%), ceftiofur (2%), ceftriaxone (1%), and nalidixic acid (1%). No isolates were resistant to amikacin, apramycin, or ciprofloxacin.

Overall, 402 (58%) *S*. Typhimurium isolates were resistant to ≥1 antimicrobial agent tested, 379 (54%) were resistant to ≥2 antimicrobial agents, and 312 (45%) were resistant to ≥5 antimicrobial agents (Table 1). Three distinct multidrug-resistant patterns were found among the 312 isolates resistant to ≥5 agents: 209 (67%) were resistant to ampicillin, chloramphenicol, streptomycin, sulfamethoxazole and tetracycline (R-type ACSSuT), 26 (8%) were additionally resistant to kanamycin (R-type ACKSSuT) and 61 (20%) were resistant to ampicillin, kanamycin, streptomycin, sulfamethoxazole, and tetracycline (R-type AKSSuT).

**Table 1:** R-type and phage type distribution among *Salmonella* Typhimurium isolates, NARMS 1997–1998

R-type^a^	All isolates N (%)	DT104 N (%)	RDNC^†^ N (%)	Untypeable N (%)
**ACSSuT**	187 (27)	160 (62)	6 (3)	6 (6)
				
ACSSuT+Cl	11 (1)	10 (4)	0	0
ACSSuT+G	2 (<1)	2 (<1)	0	0
ACSSuT+N	4 (<1)	4 (2)	0	0
ACSSuT+Tm	1 (<1)	0	0	0
ACSSuT+Cl+Cep	2 (<1)	1 (<1)	0	0
ACSSuT+Cl+Tm	1 (<1)	1 (<1)	0	0
ACSSuT+Cl+Cef+Cx+Cep	1 (<1)	1 (<1)	0	0
ACSSuT total	209 (30)	179 (69)	6 (3)	6 (6)
				
**ACKSSuT**	16 (2)	13 (5)	1 (<1)	2 (2)
				
ACKSSuT+Cep+Tm	4 (<1)	0	0	4 (4)
ACKSSuT+Cl+Cef+Cx+Cep	3 (<1)	0	0	3 (3)
ACKSSuT+Cl+Cef+Cx+Cep+G	2 (<1)	1 (<1)	0	1 (1)
ACKSSuT+Cl+Cef+Cx+Cep+G+Tm	1 (<1)	0	0	1 (1)
ACKSSuT total	26 (4)	14 (5)	1 (<1)	11 (11)
				
**AKSSuT**	51 (7)	5 (2)	8 (4)	34 (33)
				
AKSSuT+Cep	4 (<1)	1 (<1)	1 (<1)	2 (2)
AKSSuT+G	1 (<1)	0	1 (<1)	0
AKSSuT+Cl+Cep	1 (<1)	0	0	0
AKSSuT+Cep+G	2 (<1)	2 (<1)	0	0
AKSSuT+Cl+Cep+G	1 (<1)	1 (<1)	0	0
AKSSuT+Cef+Cx+Cep+N	1 (<1)	0	0	1 (1)
AKSSuT total	61 (9)	9 (4)	10 (5)	37 (36)
				
**Pansusceptible**	295 (42)	33 (13)	144 (69)	26 (25)
Resistant to 1 antimicrobial agent	23 (3)	5 (2)	11 (5)	3 (3)
Resistant to 2 antimicrobial agents	19 (3)	7 (3)	8 (4)	1 (1)
Resistant to 3 antimicrobial agents	21 (3)	4 (2)	10 (5)	4 (4)
Resistant to 4 antimicrobial agents	27 (4)	5 (2)	11 (5)	10 (10)
Resistant to 5 antimicrobial agents	6 (<1)	3 (1)	2 (1)	1 (1)
Resistant to 6 antimicrobial agents	4 (<1)	0	1 (<1)	0
Resistant to 7 antimicrobial agents	5 (<1)	0	2 (1)	1 (1)
Resistant to 9 antimicrobial agents	1 (<1)	0	0	1 (1)
Total	697 (100)	259 (100)	209 (100)	104 (100)

^a^NARMS, National Antimicrobial Resistance Monitoring System; Cl, amoxicillin-clavulanic acidl; A, ampicillin; Ceft, ceftiofur; Cx, ceftriaxone; Cep, cephalothin; C, chloramphenicol; G, .gentamicin; K, kanamycin; N, nalidixic acid; S, streptomycin; Su, sulfamethoxazole; T, tetracycline; Tm, trimethoprim-sulfamethoxazole. ^b^Reacts but does not conform.

The proportion of *S*. Typhimurium isolates that were R-type ACSSuT, R-type ACKSSuT, or R-type AKSSuT varied among the 16 NARMS sites. Among the 14 sites that submitted ≥10 *S*. Typhimurium isolates, New York State had the highest proportion of isolates that were one of these phenotypes (64%), and Minnesota had the lowest (17%) (p < 0.001) (Table 2). *S*. Typhimurium R-type ACSSuT was found in all states, and the proportion of *S*. Typhimurium isolates that were R-type ACSSuT ranged from 4% in Minnesota to 64% in New York State (Table 2). *Salmonella* Typhimurium R-type ACKSSuT isolates were not found in California, New York State, Oregon, or West Virginia. *Salmonella* Typhimurium R-type AKSSuT isolates were not found in New York State, Washington, or West Virginia, and among other sites ranged from 4% in Maryland and Colorado to 20% in Massachusetts (Table 2).

**Table 2:** *Salmonella* Typhimurium isolates with ACSSuT, ACKSSuT, or AKSSuT resistance patterns by site, NARMS 1997–1998 agent^a^

Site	ACSSuT N (%)	ACKSSuT N (%)	AKSSuT N (%)	Other R-types N (%)	Pansusceptible N (%)	Total N	
California^b^	8 (32)	0	3 (12)	5 (20)	9 (36)	25	
Colorado	16 (36)	2 (4)	2 (4)	8 (18)	17 (38)	45	
Connecticut	15 (39)	1 (3)	4 (10)	6 (15)	13 (33)	39	
Florida	4 (45)	1 (11)	1 (11)	0	3 (33)	9	
Georgia	21 (26)	1 (1)	6 (8)	9 (11)	43 (54)	80	
Kansas	2 (11)	2 (11)	1 (5)	5 (26)	9 (47)	19	
Los Angeles^c^	15 (25)	3 (5)	7 (12)	18 (31)	16 (27)	59	
Maryland	12 (44)	1 (4)	1 (4)	2 (7)	11 (41)	27	
Massachusetts	21 (24)	3 (3)	18 (21)	9 (10)	37 (42)	88	
Minnesota	2 (4)	2 (4)	5 (9)	12 (23)	32 (60)	53	
New Jersey	28 (32)	5 (6)	4 (5)	11 (13)	38 (44)	86	
New York City^d^	10 (21)	2 (4)	7 (15)	7 (15)	22 (45)	48	
New York State^e^	21 (64)	0	0	2 (6)	10 (30)	33	
Oregon	8 (30)	0	2 (7)	2 (7)	15 (56)	27	
Washington	25 (48)	3 (6)	0	7 (13)	17 (33)	52	
West Virginia	1 (14)	0	0	3 (43)	3 (43)	7	
Total	209 (30)	26 (4)	61 (9)	106 (15)	295 (42)	697	

^a^NARMS, National Antimicrobial Resistance Monitoring System. ^b^San Francisco and Alameda Counties. ^c^Los Angeles County. ^d^Five counties of New York City (Bronx, Kings, New York, Queens, Richmond). ^e^Excluding New York City.

Both R-type ACSSuT and R-type AKSSuT isolates were identified in all months of the 2-year surveillance period. In contrast, R-type ACKSSuT resistance was first noted in isolates collected during June 1997; from this point forward, R-type ACKSSuT isolates were found every month. For each year, the proportion of *S*. Typhimurium isolates that were R-type ACSSuT in the winter (January–March) was 45% (61/135), compared with 20% (41/201) in the summer (July–September) (p < 0.001).

The proportion of multidrug-resistant isolates that were R-type ACSSuT varied significantly by age (p < 0.01) for the 542 isolates for which patient’s age was known. Those from patients 40–49 years of age had the highest proportion of R-type ACSSuT isolates (41%), and those 10–19 years of age had the lowest proportion (19%).

R-type ACSSuT strains were significantly more likely to be isolated from a sterile site (p < 0.01) than from stool when compared with other R-types or with pansusceptible *S*. Typhimurium isolates. Nine percent (19/203) of R-type ACSSuT isolates were from blood compared with 3% (9/291) of pansusceptible isolates. This association was not seen with other R-types.

Of the 209 *S*. Typhimurium R-type ACSSuT isolates, 15 (7%) were also resistant to amoxicillin-clavulanic acid, 4 (2%) were resistant to nalidixic acid, 3 (1%) were resistant to cephalothin, 2 (1%) were resistant to trimethoprim, 2 (1%) were resistant to gentamicin, 1 was resistant to ceftiofur, and 1 was resistant to ceftriaxone. Of 26 *S*. Typhimurium R-type ACKSSuT isolates, 10 (39%) were resistant to cephalothin, 6 (23%) were resistant to amoxicillin-clavulanic acid, 6 (23%) were resistant to ceftiofur, 6 (23%) were resistant to ceftriaxone, 5 (19%) were resistant to trimethoprim, and 3 (12%) were resistant to gentamicin. Of the 61 *S*. Typhimurium R-type AKSSuT isolates, 9 (15%) were resistant to cephalothin, 4 (7%) were resistant to gentamicin, 2 (3%) were resistant to amoxicillin-clavulanic acid, 1 was resistant to ceftiofur, 1 was resistant to ceftriaxone, and 1 was resistant to nalidixic acid (Table 1).

Although no *S*. Typhimurium isolates were resistant to ciprofloxacin, two isolates had reduced susceptibility to ciprofloxacin (both had MIC = 0.250 μg/mL), and both isolates were R-type ACSSuT. Twelve *S*. Typhimurium isolates had ceftriaxone MICs >32 μg/mL; 11 were resistant (MIC >64 μg/mL). Nine (82%) of the 11 ceftriaxone resistant isolates were from children <18 years of age; 7 were from children <6 years of age. As a group, these 11 isolates were among the most highly resistant seen, with 8 isolates (80%) resistant to >9 antimicrobial agents; 6 were R-type ACKSSuT, 1 was R-type ACSSuT, and 1 was R-type AKSSuT.

### Phage Testing

Of the 697 *S*. Typhimurium phage typed, 259 (37%) were DT104, 209 (30%) were RDNC, 103 (15%) were untypable; 35 other phage types were identified at low frequency (<3% of total) among the remaining 126 *S*. Typhimurium isolates (Table 3). Among the 295 pansusceptible *S*. Typhimurium isolates, there were 37 different phage types; 144 (49%) were RDNC, 33 (11%) were DT104, 26 (9%) were untypable, 20 (7%) were DT46, 14 (5%) were DT10, and 10 (3%) were DT2 isolates. Among the 296 isolates that were R-type ACSSuT, ACKSSuT, or AKSSuT, seven different phage types were found; 202 (68%) were DT104, 54 (18%) were untypable, 17 (6%) were RDNC, 7 (2%) were DT12/12A, 6 (2%) were DT21, 6 (2%) were DT 110/110B, and 4 (1%) were DT120. All three of these multidrug-resistant *S*. Typhimurium isolates included some DT104 isolates; 179 (86%) of the 209 R-type ACSSuT isolates, 14 (54%) of the 26 R-type ACKSSuT isolates, and 9 (15%) of the 61 R-type AKSSuT isolates were DT104 (Figure). All four of the nalidixic acid resistant R-type ACSSuT isolates were DT104. Two other prevalent phage categories among multidrug-resistant *S*. Typhimurium isolates were RDNC (and didn’t necessarily exhibit the same lysis pattern) and untypable. Six (3%) of the R-type ACSSuT isolates, one (4%) of the R-type ACKSSuT isolates, and 10 (3%) of the R-type AKSSuT isolates were RDNC. Six (3%) of the R-type ACSSuT isolates, 11 (42%) of the R-type ACKSSuT isolates, and 37 (61%) of the R-type AKSSuT isolates were untypable. Compared with other phage types, DT104 isolates were more likely to be R-type ACSSuT (86% vs. 3%; p < 0.01), and untypable isolates were more likely to be R-type AKSSuT (61% vs. 16%; p > 0.01).

**Table 3:** *Salmonella* Typhimurium isolates by phage type, 1997–1998

Phage type	N (%)
104	168 (24)
104A	24 (3)
104B	27 (4)
104C	4 (1)
U302	36 (5)
DT104	259 (37)
RDNC	209 (30)
Untypeable	103 (15)
1	6 (1)
2	14 (2)
4A	1 (<1)
9	1 (<1)
10	14 (2)
12/12A	10 (1)
21	6 (1)
22	2 (<1)
18	1 (<1)
36	1 (<1)
38	1 (<1)
40/40 VAR	3 (<1)
41/41A	3 (<1)
46	20 (3)
66	2 (<1)
69	2 (<1)
87	1 (<1)
105	1 (<1)
106	3 (<)
107	1 (<1)
110/110B	10 (2)
114	1 (<1)
120	6 (1)
124	2 (<1)
126	5 (<1)
156	3 (<1)
160	2 (<1)
164	1 (<1)
167	1 (<1)
170	1 (<1)
U291	1 (<1)
Total	697 (100)

**Figure:**
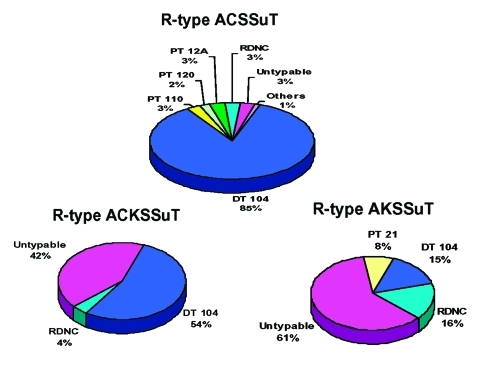
Distribution of *Salmonella* Typhimurium phage types among resistance patterns.

## Discussion

This comprehensive study of phage type and antimicrobial resistance among *Salmonella* Typhimurium isolates in the United States confirms that multidrug resistance is common among *S*. Typhimurium isolates and that DT104 is the dominant phage type. We found that 42% of *S*. Typhimurium collected during 1997–1998 belonged to one of three multidrug-resistant phenotypes: R-type ACSSuT, R-type ACKSSuT, and R-type AKSSuT. Overall, 68% of these isolates belonging to one of the multidrug-resistant phenotypes were DT104, with the greatest proportion of DT104 among the R-type ACSSuT isolates (86%); taken together, 28% of all *S*. Typhimurium isolates were DT104 R-type ACSSuT. Since an estimated 1.4 million persons are infected with *Salmonella* each year in the United States (*13*), these data suggest that approximately 100,000 persons were infected annually with *S*. Typhimurium DT104 R-type ACSSuT in 1997 and 1998 in this country.

This study also contributes to our understanding of the descriptive epidemiology of *S*. Typhimurium R-type ACSSuT. *S*. Typhimurium R-type ACSSuT isolates were found in all sites with the highest proportion from New York and the lowest from Minnesota. The proportion of *S*. Typhimurium isolates that were R-type ACSSuT increased during the winter and declined during the summer. *S*. Typhimurium R-type ACSSuT isolates were also most likely to be found in persons 40–49 years of age and least likely to be from those 10–19 years of age. *S*. Typhimurium R-type ACSSuT isolates were more likely to be isolated from sterile sites than were other multidrug-resistant or susceptible *S*. Typhimurium isolates. Further studies are needed to determine if *S*. Typhimurium R-type ACSSuT isolates are more invasive than other *S*. Typhimurium isolates.

The other two predominant multidrug-resistant *S*. Typhimurium seen were R-type ACKSSuT and AKSSuT. Those isolates were largely RDNC or untypable by phage typing. At the time phage typing was done, additional typing phages 1, 2, 3 and 18 of the Colindale scheme were not used. These additional typing phages assist in defining what would otherwise be interpreted as untypable. The untypable categories described here could include isolates that would now be designated as DT193, 194, 195, or 208 if these additional typing phages were applied. Multidrug resistance has been described among DT193 and 208 isolates identified in other studies (*14*,*15*). DT193 and 208 represented 10.9% of the pentaresistant *S.* Typhimurium from animals submitted to the U. S. National Veterinary Services Laboratory in 1998 (*15*), and DT193 was also the most prevalent phage type among 155 multidrug-resistant *S*. Typhimurium tested in southern Italy between 1992 and 1997 (*16*).

The number of phage-untypable isolates within ACKSSuT and AKSSuT resistance patterns raises the question of whether multidrug resistance is associated with untypability. Within R-type ACKSSuT, 42% of isolates were untypable and within R-type AKSSuT, 61% were untypable. Transformation of *Salmonella enterica* serotype Enteritidis (*S*. Enteritidis) with drug resistance plasmids has been documented to cause changes in phage type (*17*,*18*). Brown et al. (*17*) showed a shift of PT8 to 13a upon acquisition of IncX plasmid pOG670, which confers resistance to ampicillin and kanamycin. Threlfall et al. (*18*) reduced susceptibility to phages within the *S*. Enteritidis typing set in several *S.* Enteritidis isolates, and in two cases produced untypable isolates by transforming isolates with an IncN plasmid that contained ampicillin and streptomycin resistance factors. Further work to explore the plasmid and prophage content of isolates from each resistance type, may clarify the importance of these extrachromosomal elements in determinating resistance and phage type.

The emergence of clinically important antimicrobial resistance is cause for concern. Occurrence of third-generation cephalosporin resistance among the multidrug-resistant subpopulation of *S*. Typhimurium isolates is notable. Third-generation cephalosporins (e.g., ceftriaxone) are important in treating invasive *Salmonella* infections, particularly in children (*19*). In fact, most isolates we describe with ceftriaxone resistance came from children <18 years of age. A plasmid-mediated blaCMY-2 mechanism has been described as the source for the expanded-spectrum β-lactam resistance among *Salmonella* that includes ceftriaxone seen in recent years in the United States (*10*). The prevalence of blaCMY-2 has continued to increase among multidrug-resistant *Salmonella*, especially serotype Newport, in more recent years (*20*,*21*). Occurrence of nalidixic acid resistance among multidrug-resistant *S*. Typhimurium R-type ACSSuT DT104 is also notable. Fluoroquinolones are also important in treating invasive *Salmonella* infections, particularly in adults. Although no fluoroquinolone resistant isolates were identified in this study, four R-type ACSSuT isolates were resistant to nalidixic acid; patients with nalidixic acid resistant *Salmonella* DT104 R-type ACSSuT infections have failed treatment with fluoroquinolones (*19*).

Resistance to multiple antimicrobial agents is common among *S*. Typhimurium. With continued selective pressure that is created by antimicrobial drug use in humans, agriculture, and particularly food animals, we can expect to see a continued high prevalence of multidrug resistance among *S*. Typhimurium. Identification and subtyping of *S*. Typhimurium isolates is essential for understanding and controlling multidrug-resistant *S*. Typhimurium infections. Phage typing provides a useful, although resource-intensive, subtyping tool for this common serotype, although isolates that are RDNC and untypeable need to be categorized into groups that reflect the circulating *S*. Typhimurium strains found in the United States. Continued surveillance of antimicrobial resistance and phage types among *S*. Typhimurium will monitor dissemination of multidrug-resistant strains and strains resistant to clinically important antimicrobial agents, including cephalosporins and fluoroquinolones.
